# Supplementary material for: Pathophysiological Significance of Neutrophilic Transfer RNA-Derived Small RNAs in Asymptomatic Moyamoya Disease
Source: Cells. 2021 May 1;10(5):1086. doi: 10.3390/cells10051086 (PMC8147334; doi:10.3390/cells10051086)

2D Structure	Local AU	Position

```
717                                    742
                          8mer-1a
5'-tataGT-AGAATC-TTATAC-ACGGAAAa-3' utr
      ||  |  ||:|  :||:||  |||||||
3'-uuugCACUAUUGGUGAUGUGAUGCCUUUg-5' tRF
            16151413      7 6 5 4 3 2
           3' Pairing        Seed
```

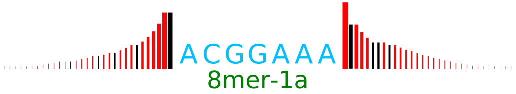

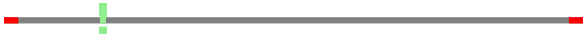

2D Structure	Local AU	Position

```
425                                    447
                          8mer-1a
5'-aaATGTGGAAA-----ACA-TACGGAAAa-3' utr
     |:|||:  ||     |||  ||||||||
3'-uuUGCACUAUUGGUGAUGUGAUGCCUUUg-5' tRF
            16151413      7 6 5 4 3 2
           3' Pairing        Seed
```

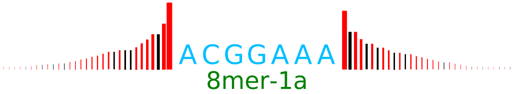

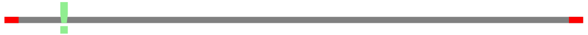

Supplement: Supplementary file 1 [file cells-10-01086-s001.zip › cells-1149161 3rd supplementary materials/Data Supplements/Data Supplement V.zip!/Data supplement 6-mRNAs targets of tsRNAs/tsRNA-Val-AAC-017/Diagrams/tRF-Val-AAC-017_vs_ENST00000360150(ERGIC2).pdf]
